# Safety of accessing brachial veins for large-bore upper extremity venous thrombectomy using ClotTriever Thrombectomy System

**DOI:** 10.1186/s42155-024-00509-8

**Published:** 2025-01-11

**Authors:** Luke A. Verst, Colvin Greenberg, David S. Shin, Matthew Abad-Santos, Eric J. Monroe, Mina S. Makary, Jeffrey Forris Beecham Chick

**Affiliations:** 1 Department of Radiology, Section of Vascular and Interventional Radiology, University of Washington, 1959 Northeast Pacific Street, Seattle, WA 98195 USA; 2 Department of Radiology, Division of Vascular and Interventional Radiology, University of Southern California, 1500 San Pablo Street, Los Angeles, CA 90033 USA; 3 Department of Radiology, Section of Vascular and Interventional Radiology, University of Wisconsin, 1675 Highland Avenue, Madison, WI 53792 USA; 4 Department of Radiology, Division of Vascular and Interventional Radiology, The Ohio State University Wexner Medical Center, Columbus, OH 43210 USA

## Abstract

**Purpose:**

To evaluate access site adverse events following ClotTriever-mediated large-bore mechanical thrombectomy via small upper extremity deep veins (< 6-mm).

**Materials and methods:**

Twenty patients, including 24 upper extremity venous access sites, underwent ClotTriever-mediated large-bore thrombectomy of the upper extremity and thoracic central veins for symptomatic deep vein obstruction unresponsive to anticoagulation. Patients without follow-up venous duplex examinations (*n* = 3) were excluded. Patients who had > 6-mm diameter veins accessed (*n* = 3) were excluded. Temporary purse-string sutures and manual pressure were used for access site hemostasis in all patients. Vein access site and diameter, technical success (defined as placement of the 13.5-French ClotTriever sheath followed by thrombectomy), and early (< 30-days) and late (> 30-days) access site-related adverse events (according to the Adverse Event Classification by the *Society of Interventional Radiology* criteria) were recorded.

**Results:**

Fourteen patients (8 males, 6 females; mean age 51.7 ± 13.6 years) comprising 16 upper extremity venous access sites were included in this study. Access sites included: right brachial (*n* = 7), left brachial (*n* = 5), and bilateral brachial (*n* = 2) veins. The mean access site diameter was 4.3-mm ± 0.67-mm. Technical success was achieved via all access sites. Six (42.9%) patients underwent stent reconstruction following thrombectomy through the same accesses. After the procedure, all purse-string sutures were removed within 24 h. Three (21.4%) patients experienced small access site hematomas that did not require transfusion, intervention, or prolonged hospitalization. Initial follow-up venous duplex ultrasounds were performed at 29.3 ± 21.7 days following intervention. The mean follow-up interval to the second and third venous duplex ultrasounds were 124.3 ± 64-days and 225.1 ± 80.1 days, respectively. One (7.1%) patient developed right arm swelling six days after the procedure and was found to have thrombosis of the previously accessed right brachial vein. No other clinically or sonographically significant access site adverse events were observed.

**Conclusion:**

ClotTriever-mediated large-bore thrombectomy via small upper extremity veins is safe with minimal access site adverse events.

## Introduction

Upper extremity (UE) deep vein thrombosis (DVT) has increased in prevalence over the past decade and is now estimated to constitute 10% of acute DVTs [1]. Clinical outcomes from adverse events of UE DVT such as thrombophlebitis, pulmonary embolism (PE), and post-thrombotic syndrome (PTS) mirror those of lower extremity DVT [2]. Despite the prevalence of UE DVT, there remain no established treatment guidelines. Current management practices are often determined by institutional preference and may involve anticoagulation, catheter-directed thrombolysis, and percutaneous mechanical thrombectomy [3–5].

Use of the ClotTriever Thrombectomy System (Inari Medical; Irvine, CA) is increasing in the treatment of caval and lower extremity DVT [6–8]. There are increased instances of its use in treating symptomatic deep venous occlusive disease of the UE in patients for whom pharmacological thrombolysis is contraindicated [4, 5, 9–11]. The use of ClotTriever in UE DVT has been limited due to its large caliber (13.5-French (F)) and the manufacturer’s recommendation against its use in veins smaller than 6-mm in diameter. The safety of short-term, large-bore access into small veins (< 6-mm) has not yet been established. This study aims to evaluate the technical feasibility and access-site related adverse events following the use of the ClotTriever Thrombectomy System to treat UE DVT via small vein access sites.

## Materials and methods

### Study design

This single-center, retrospective, and descriptive study was conducted under institutional review board (IRB) approval and complied with the Health Insurance Portability and Accountability Act (HIPAA). The need for informed consent was exempt by the IRB due to the retrospective design.

### Patient selection and diagnostic imaging

Twenty patients underwent endovascular intervention for symptomatic deep venous occlusive disease of the UE deep veins and thoracic central veins using the ClotTriever system (Inari Medical) with its 13.5-F sheath. In this cohort of twenty patients, three patients were excluded due to lack of follow-up, and three patients were excluded due to access vein diameter > 6-mm. Fourteen patients underwent thrombectomy via access into a small deep vein, defined as < 6-mm in diameter. All fourteen patients underwent preprocedural computed tomography venography (CTV) of the chest and/or UE duplex venous ultrasound. No prior venous interventions had been performed on these patients.

### Procedural technique

**Fig. 1:**
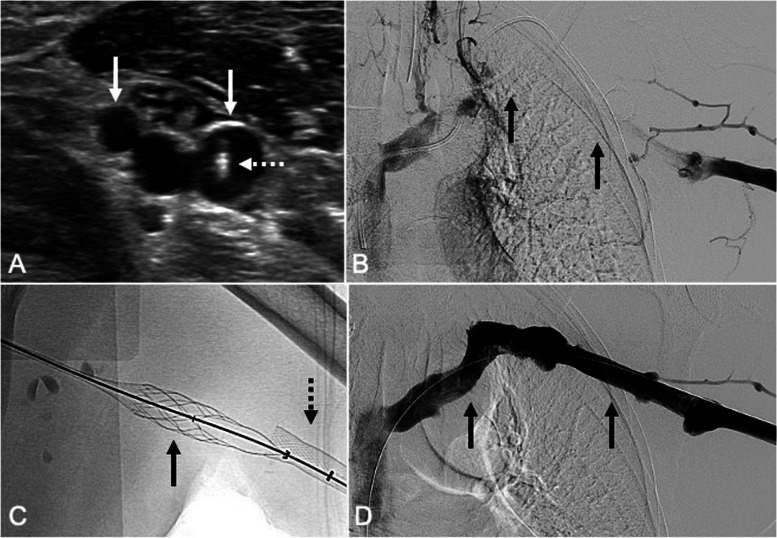
A 54-year-old man with breast cancer post right mastectomy undergoing chemotherapy via a left-sided chest port presented with left arm swelling after playing tennis. **A** Ultrasound-guided access of one of the paired brachial veins (solid arrows) was obtained using a 21-gauge micropuncture needle (dashed arrow). **B** Left upper extremity venography demonstrated occlusion of the left brachiocephalic, subclavian, and axillary veins (solid arrows). **C** Thrombectomy of the left upper extremity veins was performed with the 13.5-French ClotTriever Thrombectomy System (solid arrow) after upsizing the access sheath to the funnel-tipped ClotTriever Sheath (dashed arrow). **D** Completion left upper extremity venography demonstrated brisk in-line flow from the left brachial vein to the right atrium (solid arrows)

The procedures were completed with either conscious sedation or general anesthesia. Intravenous heparin was administered during the interventions, starting with a 100 units/kg bolus followed by additional boluses of 2000–3000 units as needed, at the operator’s discretion. A representative procedure is shown in Fig. 1. UE access sites were assessed with real-time ultrasound and measured without the presence of a tourniquet. Brachial vein access in the upper half of the arm was used in all cases and obtained with ultrasound guided micropuncture technique. The thoracic central veins were recanalized using either blunt or sharp techniques, which have been previously described [12–17]. After establishing wire access to the inferior vena cava, mechanical thrombectomy was performed using the ClotTriever Thrombectomy System (Inari Medical). One to four passes with the device were performed through each access site.

In some cases, venoplasty and stent reconstruction were necessary to address underlying venous stenosis, as previously described [18]. After the procedure, completion venography and intravascular ultrasound (IVUS) were performed to evaluate the final technical outcomes. Temporary purse-string sutures and manual pressure were used for hemostasis at the access site. All sutures were removed within 24 h.

### Post-procedure management and follow-up

Following intervention, all patients were admitted for observation and initiation of a heparin infusion. After post-procedure day one, patients were transitioned from heparin to 1 mg/kg enoxaparin twice daily for 6–12 months. Patients who underwent thrombectomy and venous reconstruction with stenting were also prescribed 81-mg of aspirin daily. Any deviations from this anticoagulation regimen were decided on a case-by-case basis. Technical success was defined as the successful placement and use of the ClotTriever Thrombectomy System, resulting in the extraction of thrombotic materials on visual inspection and a reduction in thrombus burden as shown on repeat venography. Clinical success was determined based on the patient's reported improvement of presenting symptoms at clinical follow-up with concurrent bilateral UE venous duplex examinations.

## Results

### Patient demographics and presenting symptoms

Fourteen patients (8 males, 6 females; 51.7 ± 13.6 years) underwent ClotTriever-mediated thrombectomy of the UE deep veins and central thoracic veins via brachial veins measuring < 6-mm. Presenting symptoms included: UE and/or facial swelling (*n* = 14), UE pain (*n* = 6), fever (*n* = 1), and dyspnea (*n* = 1). Eleven (78.6%) patients had benign occlusions, and three (21.4%) patients had malignant occlusions. Ten (71.4%) patients had acute DVT, and four (28.6%) patients had chronic DVT.

### Technical details and outcomes

*Venous access sites and their respective diameters are outlined in *Table 1. The mean access site diameter was 4.3-mm ± 0.67-mm. A 13.5-F ClotTriever Sheath was used in all patients. One-to-four thrombectomy sweeps were made via each access site. Procedural technical success, defined as successful venous access and subsequent thrombectomy, was achieved at all access sites (100%). Six (42.9%) patients underwent additional venous stent reconstruction following thrombectomy to address the underlying venous occlusive disease. In patients undergoing stent reconstruction, the mean number of stents deployed was 3.4 (range, 2–6 stents), with a mean diameter of 11.8-mm ± 1.6-mm (range, 10–14-mm). No patients required catheter-directed thrombolysis or intensive care admission. Access site purse-string sutures were removed within 24 h.

**Table 1:** Venous access sites and their respective diameters as visualized on intraprocedural ultrasound

Patient	Access Site(s)	Accessed Vein Diameter(s) (mm)
1	Right Brachial	3.4
2	Right brachial	5.8
3	Left Brachial	3.8
4	Right Brachial	4.1
5	Right Brachial	4.0
6	Right Brachial	3.6
7	Left Brachial	4.4
8	Left Brachial	3.8
9	Right Brachial	3.7
10	Left Brachial	5.3
11	Right Brachial	3.9
12	Bilateral Brachial	4.1, 4.8
13	Left Brachial	4.4
14	Bilateral Brachial	5.1, 4.5

### Imaging and clinical outcomes

Twenty-eight follow-up venous duplex ultrasounds were performed with a mean of two ultrasounds/patient. The mean time to the first venous duplex ultrasound was 29.3 ± 21.7 days. The mean time to the second and third venous duplex ultrasound was 124.3 ± 64 days and 225.1 ± 80.1-days, respectively. Three patients were found to have recurrent thrombosis of the previously treated venous segments on follow up imaging, one of whom experienced symptomatic thrombosis of the accessed brachial vein. One patient had recurrent thrombosis of the superior vena cava 124 days post procedure which was treated with mechanical thrombectomy. The second patient was found to have recurrent thrombosis of the right axillary vein 27 days post procedure and was not treated given hospice disposition and absence of reported symptoms. The third patient experienced right UE swelling six days post procedure and was found to have recurrent thrombosis of the right axillary, innominate, and subclavian veins with involvement of the right brachial vein. Follow-up ultrasound at 250 days demonstrated persistent thrombosis of the single affected right brachial vein extending into the right axillary vein. The subclavian vein thrombus resolved and the patient was no longer symptomatic while remaining on apixaban and aspirin. No patients underwent surgical decompression.

### Adverse events

There were no intraprocedural adverse events, including clinically significant cardiac tamponade or arrhythmia. No post-procedural hemorrhage, stroke or pulmonary embolism occurred. There were no cases of post-procedural UE arterial insufficiency or neurovascular compromise. Three (21.4%) patients experienced asymptomatic mild brachial vein access site hematomas that did not require transfusion or escalation of care. One (7.1%) patient developed right brachial access site thrombosis six days after intervention. Following SIR standards, there were three (21.4%) Class A minor immediate adverse events (hematomas), and one (7.1%) Class A long-term adverse event (brachial vein access site thrombosis).

## Discussion

Mechanical thrombectomy for benign and malignant thoracic central venous occlusions has been shown to be technically feasible and clinically efficacious with low adverse event rates in patients for whom thrombolysis is contraindicated [7, 19, 20]. The feasibility and safety of using large-bore mechanical thrombectomy devices via small vein access in the UE is unknown.

Successful ClotTriever-mediated thrombectomy of the iliofemoral system via popliteal vein access has been previously reported without hematomas or perforations of the accessed popliteal veins [21, 22]. Cadaveric studies have described the normal diameter of the popliteal veins at 5–12-mm in females, 7–13-mm in males, and 5–7-mm in adults < 30 years [23, 24].

Currently, no reports exist in the literature assessing small (< 6-mm) venous access site adverse events including hematoma or neuropraxia after large-bore (8-F or larger) UE thrombectomy. Prior literature has demonstrated a significant positive correlation between rates of UE deep and superficial vein thrombosis, and peripherally inserted central catheter (PICC) diameters [10, 11, 25, 26]. However, sustained obstruction to venous flow in the setting of an indwelling catheter, active chemotherapy infusion, inpatient status, and sepsis or inflammation are confounding risk factors for UE DVT in the period following venous access placement [27]. Ultrasound-guided access for single-stick or atraumatic access to the vein, temporary sheath dwell time, and procedural anticoagulation are used to mitigate risk factors that are otherwise associated with DVT development [28].

In this study, small venous access for ClotTriever-mediated thrombectomy allowed for successful thrombectomy without major access site adverse events. One patient was found to have long-term thrombosis of the accessed brachial vein. All small venous access attempts with the device were technically successful even as the device size (4.6-mm) exceeded the vessel size in some cases.

Limitations of this paper include its small sample size, single-institution nature, and retrospective design. Variables including comorbid risk factors for UE catheter-associated DVT, duration of peri-procedural anticoagulation, cancer type (if present), and the presence of previous DVT or thrombophlebitis were not controlled. Further stratification of these variables may help identify future patients likely to develop complications related to large bore access.

## Conclusions

Large-bore venous access of small (< 6-mm) UE veins is both feasible and safe, enabling the treatment of symptomatic UE and thoracic central DVT with the ClotTriever system (13.5-F sheath).
